# A comparative transcriptomic dataset of hepatocytes derived from a panel of human induced pluripotent stem cells with different ethnicities as an *in vitro* population variability toxicity model

**DOI:** 10.1016/j.dib.2026.113350

**Published:** 2026-10-12

**Authors:** Xiugong Gao, Miranda R. Yourick, Rong Li, Kayla Campasino, Robert L. Sprando, Jeffrey J. Yourick

**Affiliations:** Division of Toxicology, Office of Chemistry and Toxicology, Office of Laboratory Operations and Applied Science, Human Foods Program, U.S. Food and Drug Administration, Laurel, MD, 20708, USA

**Keywords:** In vitro model, Population variability, Ethnicity, Hepatotoxicity, Hepatocytes, Induced pluripotent stem cell, Diglycolic acid, Usnic acid

## Abstract

Interindividual differences in response to chemicals have been typically addressed through the use of a 10-fold default “uncertainty” factor. It was only recently that *in vitro* models emerged to quantitatively assess interindividual variability in the human population for specific chemicals. We have attempted to establish an *in vitro* model for assessing population variability in hepatotoxicity testing using a panel of hepatocytes derived from nine human induced pluripotent stem cell (iPSC) lines belonging to three different ethnic groups, Black or African American (BAA), Latino or Hispanic (LOH), and Non-Hispanic White (NHW). The dataset described here was collected from the panel of iPSC-derived hepatocytes with or without exposure to two chemicals, diglycolic acid (DGA) and usnic acid (UA). Cells differentiated on day 23 were exposed to 3.0 mM DGA or 10.0 μM UA for 24 h and total RNA were isolated from the cells. Global gene expression was profiled by RNA sequencing (RNA-seq) on the Illumina NextSeq 2000 platform. Two replicates (n = 2) were included for each treatment group including untreated (control) cells. The RNA-seq dataset has been deposited in the Gene Expression Omnibus (GEO; http://www.ncbi.nlm.nih.gov/geo/) repository of the National Center for Biotechnology Information with accession number GSE293032. The dataset provides a baseline for assessing interindividual variability in transcriptomic profile as well as in response to chemical exposure among the human population.

Specifications Table

**Table:** 

Subject	Biology
Specific subject area	Transcriptomic profiling of human iPSC-derived hepatocytes with different ethnicities to assess population variability in chemical toxicity
Type of data	RNA-seq data: raw data in *.fastq* format and processed data in *.quant.genes.sf* format (transcript quantification at the gene level)
Data collection	RNA-seq was conducted on an Illumina NextSeq 2000 instrument. Sequencing libraries were prepared using the Illumina Stranded mRNA Prep, Ligation kit following protocols provided by the manufacturer. Concatenated base call files (*.cbcl*) generated from the sequencing run were converted to a *.fastq* file for each sample by the DRAGEN BCL Convert pipeline. The *.cbcl* files were further analyzed by the DRAGEN RNA pipeline to generate transcript quantification results at the gene level (*.quant.genes.sf*).
Data source location	U.S. Food and Drug Administration Laurel, MD USA
Data accessibility	Repository name: Gene Expression Omnibus (GEO) Data identification number: GSE293032 Direct URL to data: https://www.ncbi.nlm.nih.gov/geo/query/acc.cgi?acc=GSE293032
Related research article	Gao X, Li R, Campasino K, Yourick MR, Zhao Y, Sprando RL, Yourick JJ. Hepatocytes derived from human induced pluripotent stem cells: Towards establishing an in vitro model for assessing population variability in hepatotoxicity testing. Curr Res Toxicol 2025; 8: 100,238. doi: 10.1016/j.crtox.2025.100238.

## 1 Value of the Data

• The data provide baseline interindividual comparison of hepatocyte transcriptomic profiling.
• The data also provide assessment of interindividual variability in toxicity response to chemicals.
• The data are potentially useful for analyzing ethnicity-specific hepatotoxic effects of chemical exposures.
• The data may be of interest to the general biomedical research community and may be especially beneficial to researchers studying human population variability.

## 2 Background

For decades, regulators have relied on a default 10-fold “uncertainty” factor to account for interindividual differences in human risk assessment when data are limited [1]. While generally reasonable, evidence shows that variability can exceed this default for some chemicals [2], highlighting the need for predictive models that more accurately quantify human variability. One strategy uses *in vitro* models with cells from multiple individuals [1]. More recently, iPSC-derived cells, especially cardiomyocytes, have enabled population-based toxicity studies [3]. Because the liver is central to chemical metabolism, models for hepatotoxicity are especially important but underdeveloped due to technical challenges in generating hepatocytes [4]. In a previous study, we attempted to establish an *in vitro* model for assessing population variability in toxicity testing using a panel of hepatocytes derived from human iPSC lines with different ethnicities [5]. The dataset compiled here not only include transcriptomics data for baseline comparison among the individual cell lines, as described in the published article [5], but also include data on transcriptomic changes in these cell lines upon exposure to two chemicals, diglycolic acid (DGA) and usnic acid (UA). DGA is an important industrial chemical and an indirect food additive and is notably recognized as the primary toxic metabolite responsible for kidney injury following diethylene glycol (DEG) poisoning [6,7]. DGA also accumulates in the liver and directly induces hepatotoxicity through mitochondrial dysfunction and cellular necrosis [[6], [7], [8]]. UA is a naturally occurring dibenzofuran derivative found primarily in several lichen species, including *Usnea* and *Cladonia*, and has been used as an antimicrobial agent and preservative in cosmetics, toothpastes, and topical creams [9]. UA was also widely marketed as a dietary supplement for weight loss and bodybuilding but was subsequently withdrawn from the market following numerous reports of acute liver injury and liver failure [10,11]. Its hepatotoxicity is thought to result primarily from mitochondrial uncoupling and oxidative stress induced directly by the parent compound [9,[12], [13], [14]].

## 3 Data Description

The dataset consists of nine cell lines (B1, B2, B3; L1, L2, L3; W1, W2, W3) belonging to three ethnicity groups: Black or African American (BAA), Latino or Hispanic (LOH), and Non-Hispanic White (NHW). Three treatment conditions applied to each cell line: control, DGA treated, or UA treated. Each experimental condition has two biological replicates, biol rep1 and biol rep2. Therefore, a total of 54 samples were contained in the dataset (Table 1).

**Table 1:** List of samples in the dataset.

Sample	Cell line	Ethnicity^a^	Treatment b	Processed data file	Raw data file 1	Raw data file 2
B1, control, biol rep1	B1	BAA	control	B1_CTL_01.quant.genes.sf	B1_CTL_01_R1.fastq	B1_CTL_01_R2.fastq
B1, control, biol rep2	B1	BAA	control	B1_CTL_02.quant.genes.sf	B1_CTL_02_R1.fastq	B1_CTL_02_R2.fastq
B1, DGA treated, biol rep1	B1	BAA	DGA	B1_DGA_01.quant.genes.sf	B1_DGA_01_R1.fastq	B1_DGA_01_R2.fastq
B1, DGA treated, biol rep2	B1	BAA	DGA	B1_DGA_02.quant.genes.sf	B1_DGA_02_R1.fastq	B1_DGA_02_R2.fastq
B1, UA treated, biol rep1	B1	BAA	UA	B1_UA_01.quant.genes.sf	B1_UA_01_R1.fastq	B1_UA_01_R2.fastq
B1, UA treated, biol rep2	B1	BAA	UA	B1_UA_02.quant.genes.sf	B1_UA_02_R1.fastq	B1_UA_02_R2.fastq
B2, control, biol rep1	B2	BAA	control	B2_CTL_01.quant.genes.sf	B2_CTL_01_R1.fastq	B2_CTL_01_R2.fastq
B2, control, biol rep2	B2	BAA	control	B2_CTL_02.quant.genes.sf	B2_CTL_02_R1.fastq	B2_CTL_02_R2.fastq
B2, DGA treated, biol rep1	B2	BAA	DGA	B2_DGA_01.quant.genes.sf	B2_DGA_01_R1.fastq	B2_DGA_01_R2.fastq
B2, DGA treated, biol rep2	B2	BAA	DGA	B2_DGA_02.quant.genes.sf	B2_DGA_02_R1.fastq	B2_DGA_02_R2.fastq
B2, UA treated, biol rep1	B2	BAA	UA	B2_UA_01.quant.genes.sf	B2_UA_01_R1.fastq	B2_UA_01_R2.fastq
B2, UA treated, biol rep2	B2	BAA	UA	B2_UA_02.quant.genes.sf	B2_UA_02_R1.fastq	B2_UA_02_R2.fastq
B3, control, biol rep1	B3	BAA	control	B3_CTL_01.quant.genes.sf	B3_CTL_01_R1.fastq	B3_CTL_01_R2.fastq
B3, control, biol rep2	B3	BAA	control	B3_CTL_02.quant.genes.sf	B3_CTL_02_R1.fastq	B3_CTL_02_R2.fastq
B3, DGA treated, biol rep1	B3	BAA	DGA	B3_DGA_01.quant.genes.sf	B3_DGA_01_R1.fastq	B3_DGA_01_R2.fastq
B3, DGA treated, biol rep2	B3	BAA	DGA	B3_DGA_02.quant.genes.sf	B3_DGA_02_R1.fastq	B3_DGA_02_R2.fastq
B3, UA treated, biol rep1	B3	BAA	UA	B3_UA_01.quant.genes.sf	B3_UA_01_R1.fastq	B3_UA_01_R2.fastq
B3, UA treated, biol rep2	B3	BAA	UA	B3_UA_02.quant.genes.sf	B3_UA_02_R1.fastq	B3_UA_02_R2.fastq
L1, control, biol rep1	L1	LOH	control	L1_CTL_01.quant.genes.sf	L1_CTL_01_R1.fastq	L1_CTL_01_R2.fastq
L1, control, biol rep2	L1	LOH	control	L1_CTL_02.quant.genes.sf	L1_CTL_02_R1.fastq	L1_CTL_02_R2.fastq
L1, DGA treated, biol rep1	L1	LOH	DGA	L1_DGA_01.quant.genes.sf	L1_DGA_01_R1.fastq	L1_DGA_01_R2.fastq
L1, DGA treated, biol rep2	L1	LOH	DGA	L1_DGA_02.quant.genes.sf	L1_DGA_02_R1.fastq	L1_DGA_02_R2.fastq
L1, UA treated, biol rep1	L1	LOH	UA	L1_UA_01.quant.genes.sf	L1_UA_01_R1.fastq	L1_UA_01_R2.fastq
L1, UA treated, biol rep2	L1	LOH	UA	L1_UA_02.quant.genes.sf	L1_UA_02_R1.fastq	L1_UA_02_R2.fastq
L2, control, biol rep1	L2	LOH	control	L2_CTL_01.quant.genes.sf	L2_CTL_01_R1.fastq	L2_CTL_01_R2.fastq
L2, control, biol rep2	L2	LOH	control	L2_CTL_02.quant.genes.sf	L2_CTL_02_R1.fastq	L2_CTL_02_R2.fastq
L2, DGA treated, biol rep1	L2	LOH	DGA	L2_DGA_01.quant.genes.sf	L2_DGA_01_R1.fastq	L2_DGA_01_R2.fastq
L2, DGA treated, biol rep2	L2	LOH	DGA	L2_DGA_02.quant.genes.sf	L2_DGA_02_R1.fastq	L2_DGA_02_R2.fastq
L2, UA treated, biol rep1	L2	LOH	UA	L2_UA_01.quant.genes.sf	L2_UA_01_R1.fastq	L2_UA_01_R2.fastq
L2, UA treated, biol rep2	L2	LOH	UA	L2_UA_02.quant.genes.sf	L2_UA_02_R1.fastq	L2_UA_02_R2.fastq
L3, control, biol rep1	L3	LOH	control	L3_CTL_01.quant.genes.sf	L3_CTL_01_R1.fastq	L3_CTL_01_R2.fastq
L3, control, biol rep2	L3	LOH	control	L3_CTL_02.quant.genes.sf	L3_CTL_02_R1.fastq	L3_CTL_02_R2.fastq
L3, DGA treated, biol rep1	L3	LOH	DGA	L3_DGA_01.quant.genes.sf	L3_DGA_01_R1.fastq	L3_DGA_01_R2.fastq
L3, DGA treated, biol rep2	L3	LOH	DGA	L3_DGA_02.quant.genes.sf	L3_DGA_02_R1.fastq	L3_DGA_02_R2.fastq
L3, UA treated, biol rep1	L3	LOH	UA	L3_UA_01.quant.genes.sf	L3_UA_01_R1.fastq	L3_UA_01_R2.fastq
L3, UA treated, biol rep2	L3	LOH	UA	L3_UA_02.quant.genes.sf	L3_UA_02_R1.fastq	L3_UA_02_R2.fastq
W1, control, biol rep1	W1	NHW	control	W1_CTL_01.quant.genes.sf	W1_CTL_01_R1.fastq	W1_CTL_01_R2.fastq
W1, control, biol rep2	W1	NHW	control	W1_CTL_02.quant.genes.sf	W1_CTL_02_R1.fastq	W1_CTL_02_R2.fastq
W1, DGA treated, biol rep1	W1	NHW	DGA	W1_DGA_01.quant.genes.sf	W1_DGA_01_R1.fastq	W1_DGA_01_R2.fastq
W1, DGA treated, biol rep2	W1	NHW	DGA	W1_DGA_02.quant.genes.sf	W1_DGA_02_R1.fastq	W1_DGA_02_R2.fastq
W1, UA treated, biol rep1	W1	NHW	UA	W1_UA_01.quant.genes.sf	W1_UA_01_R1.fastq	W1_UA_01_R2.fastq
W1, UA treated, biol rep2	W1	NHW	UA	W1_UA_02.quant.genes.sf	W1_UA_02_R1.fastq	W1_UA_02_R2.fastq
W2, control, biol rep1	W2	NHW	control	W2_CTL_01.quant.genes.sf	W2_CTL_01_R1.fastq	W2_CTL_01_R2.fastq
W2, control, biol rep2	W2	NHW	control	W2_CTL_02.quant.genes.sf	W2_CTL_02_R1.fastq	W2_CTL_02_R2.fastq
W2, DGA treated, biol rep1	W2	NHW	DGA	W2_DGA_01.quant.genes.sf	W2_DGA_01_R1.fastq	W2_DGA_01_R2.fastq
W2, DGA treated, biol rep2	W2	NHW	DGA	W2_DGA_02.quant.genes.sf	W2_DGA_02_R1.fastq	W2_DGA_02_R2.fastq
W2, UA treated, biol rep1	W2	NHW	UA	W2_UA_01.quant.genes.sf	W2_UA_01_R1.fastq	W2_UA_01_R2.fastq
W2, UA treated, biol rep2	W2	NHW	UA	W2_UA_02.quant.genes.sf	W2_UA_02_R1.fastq	W2_UA_02_R2.fastq
W3, control, biol rep1	W3	NHW	control	W3_CTL_01.quant.genes.sf	W3_CTL_01_R1.fastq	W3_CTL_01_R2.fastq
W3, control, biol rep2	W3	NHW	control	W3_CTL_02.quant.genes.sf	W3_CTL_02_R1.fastq	W3_CTL_02_R2.fastq
W3, DGA treated, biol rep1	W3	NHW	DGA	W3_DGA_01.quant.genes.sf	W3_DGA_01_R1.fastq	W3_DGA_01_R2.fastq
W3, DGA treated, biol rep2	W3	NHW	DGA	W3_DGA_02.quant.genes.sf	W3_DGA_02_R1.fastq	W3_DGA_02_R2.fastq
W3, UA treated, biol rep1	W3	NHW	UA	W3_UA_01.quant.genes.sf	W3_UA_01_R1.fastq	W3_UA_01_R2.fastq
W3, UA treated, biol rep2	W3	NHW	UA	W3_UA_02.quant.genes.sf	W3_UA_02_R1.fastq	W3_UA_02_R2.fastq

a BAA, Black or African American; LOH, Latino or Hispanic; NHW, Non-Hispanic White.

b DGA, diglycolic acid; UA, usnic acid.

The sequencing run generated a total of 173.01 GB raw data, with 89.86% ≥Q30. Detailed quality metrics of the RNA-seq data is shown in Fig. 1. The number of identified reads for the samples (indexes) were demonstrated in Fig. 2. For each sample, one processed data file in *.quant.genes.sf* format (transcript quantification at the gene level) was deposited. In addition, two raw data files (in *.fastq* format) were also included, one for forward reading (R1), the other for reverse reading (R2). Fig. 3 displays unsupervised data exploration of the processed data using principal component analysis (PCA). The PCA plot shows that the samples were clustered by individual cell lines. Treatments with DGA or UA had relatively less impacts on overall gene expression compared to interindividual variabilities. In most cases, the two replicates of each condition agreed well with each other.

**Fig. 1:**
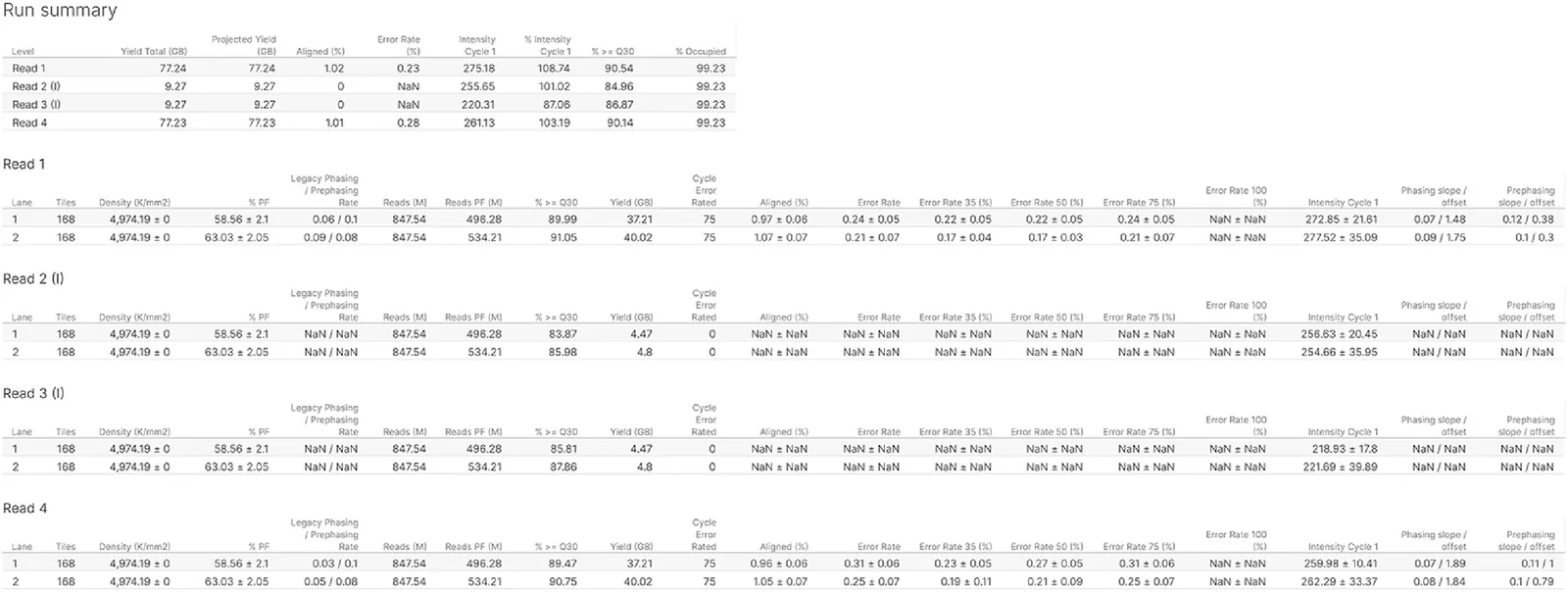
Detailed quality metrics of the RNA-seq run. The sequencing was performed on an Illumina NextSeq 2000 instrument with a P3 200-cycle flow cell. Paired 75 bp reads were generated for the samples. Reads 1 and 4 were generated from forward and reverse strands respectively; while Reads 2 and 3 were from i7 and i5 index reads respectively. The run summary was generated using Illumina Sequencing Analysis Viewer (SAV) V3.0.0.

**Fig. 2:**
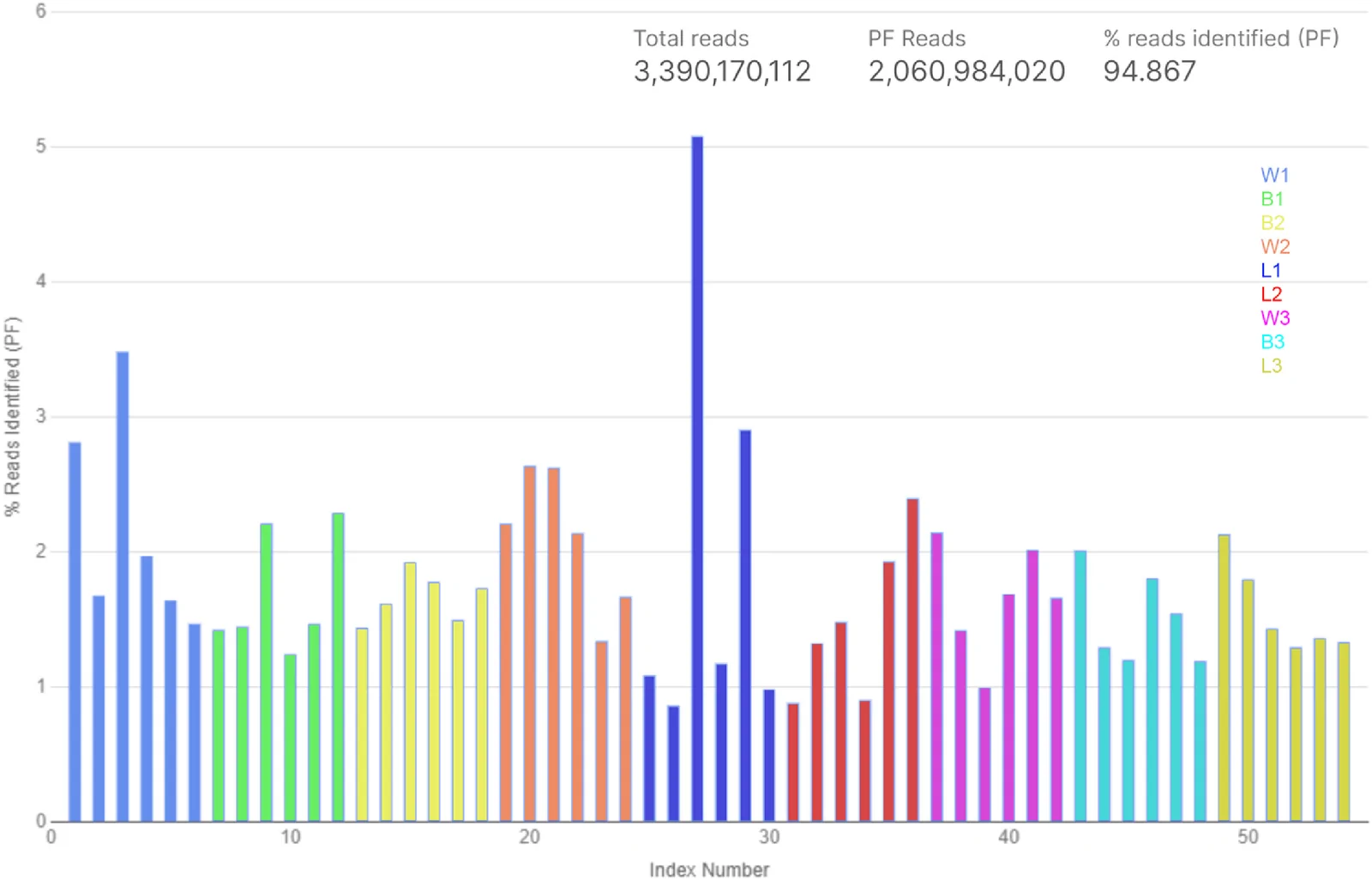
Reads mapped to each of the 54 indexes (samples). The number of *total reads, PF reads* and *% reads identified (PF)* are indicated at the top right corner of the figure. *PF reads* is the total number of passing filter reads; % reads identified (PF) is the total fraction of passing filter reads assigned to an index. The plot was generated using Illumina Sequencing Analysis Viewer (SAV) V3.0.0 and modified. The samples are color-coded by cell line as indicated in the legend. The six samples of each cell line were in the order of: control_biol rep1, control_biol rep2, DGA treated_biol rep1, DGA treated_biol rep2, UA treated_biol rep1, and UA treated_biol rep2.

**Fig. 3:**
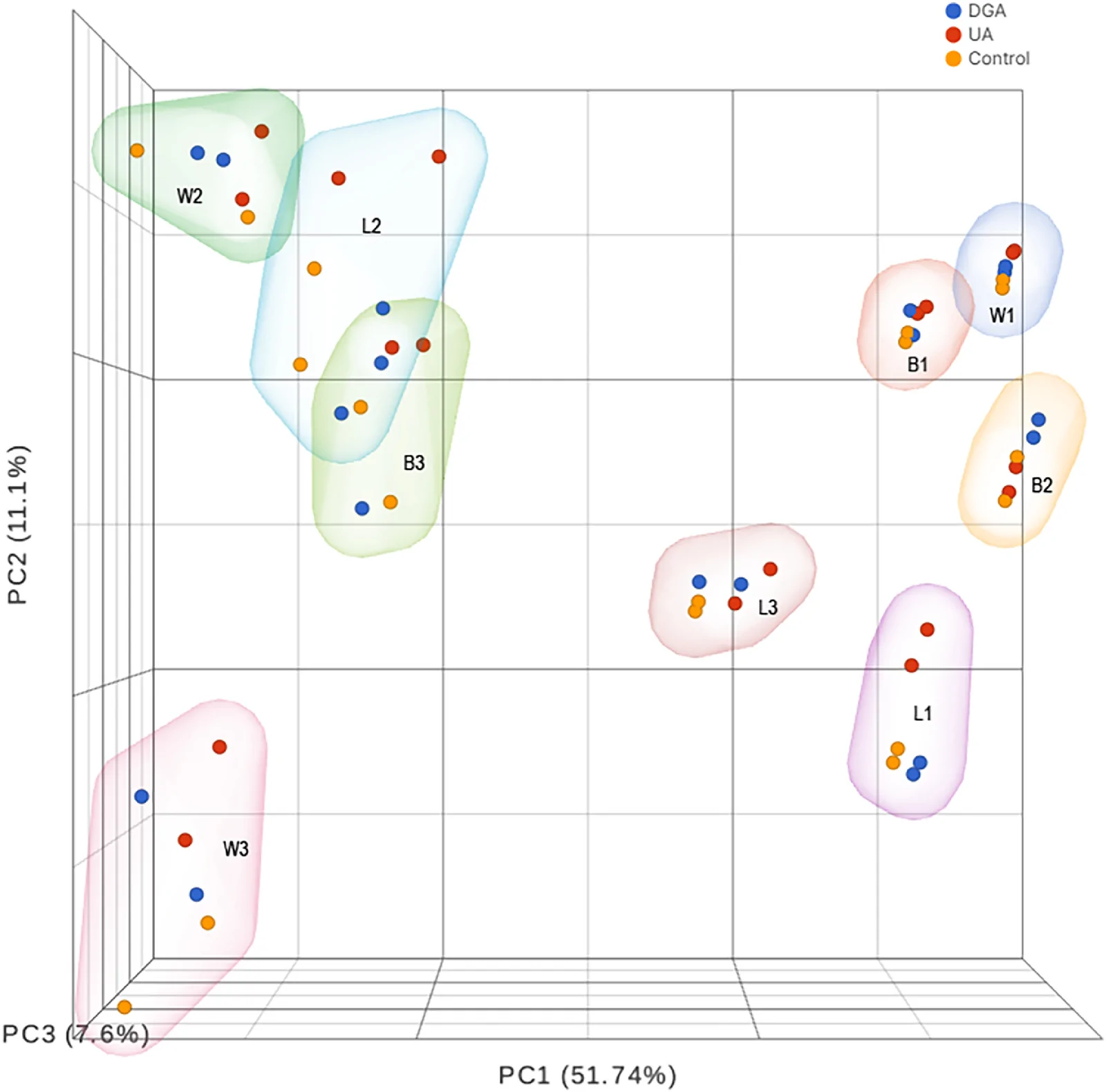
Principal component analysis (PCA) of the 54 samples in the dataset. The 6 samples from each individual cell line (color-coded for DGA, UA, and control as shown at the top-right corner; *n* = 2) were clustered in balloons labeled respectively with each cell line name. Gene-level counts for each sample were normalized using the DESeq2 median-of-ratios method and then filtered to exclude genes with a maximum normalized count ≤10 across all samples prior to PCA.

## 4 Experimental Design, Materials and Methods

### 4.1 Human iPSC lines and cell culture

Source and donor Information of the nine iPSC lines are provided in Table 2. Two of the lines (B3 and L3) were obtained from California Institute for Regenerative Medicine (CIRM; South San Francisco, CA). The remaining lines were generated in-house using endothelial progenitor cells derived from umbilical cord blood or adult peripheral blood, and the reprogramming methods have been published previously [[15], [16], [17]].

**Table 2:** List of 9 iPSC lines used in the study including donor information (gender, age, and ethnicity).

Line Name	Source	Original Name	Gender	Age	Ethnicity^b^
B1	In-house	S09 (OARSAi003-A)	Male	0	BAA
B2	In-house	S10 (OARSAi004-A)	Male	0	BAA
B3	CIRM^a^	CW20139	Male	12	BAA
L1	In-house	S14 (OARSAi007-A)	Male	23	LOH
L2	In-house	S18 (OARSAi008-A)	Male	27	LOH
L3	CIRM^a^	CW20242	Male	8	LOH
W1	In-house	S04 (OARSAi001-A)	Male	0	NHW
W2	In-house	S11 (OARSAi005-A)	Male	0	NHW
W3	In-house	S20 (OARSAi009-A)	Male	0	NHW

a CIRM, California Institute for Regenerative Medicine.

b BAA, Black or African American; LOH, Latino or Hispanic; NHW, Non-Hispanic White.

Cells were maintained as single-cell cultures (no colony formation) on COAT-1 pre-coated 6-well tissue culture plates in the Cellartis DEF-CS Culture System (Takara Bio USA, San Jose, CA) at 37 °C in a humidified incubator supplied with 5% CO_2_. For passaging, cells were dissociated into single cells with TrypLE Select Enzyme (1 ×) (Thermo Fisher Scientific, Waltham, MA) and seeded at a density of 4–5 × 10^4^ cells/cm^2^. The cell culture medium was changed daily until the cells reached a confluence of ∼1.5–3.0 × 10^5^ cells/cm^2^, which generally occurred 3–4 days post passaging. The pluripotency of the iPSCs was routinely assessed using immunocytochemistry staining of major pluripotency protein markers, including octamer-binding transcription factor 4 (OCT4), SRY (sex determining region Y)-box (SOX)2, stage-specific embryonic antigen-4 (SSEA4) and T cell receptor α locus (TRA-1–60) using a Pluripotent Stem Cell 4-Marker Immunocytochemistry Kit (cat. no. A24881) according to the manufacturer's protocol (Thermo Fisher Scientific).

### 4.2 Hepatocyte differentiation

Hepatocyte differentiation from the iPSC lines were conducted using the Cellartis iPS Cell to Hepatocyte Differentiation System (Takara Bio USA), and instructions from the manufacturer were followed. First, iPSCs were guided to differentiate into definitive endoderm (DE) using the Cellartis Definitive Endoderm Differentiation Kit. Briefly, on day 0, 6-well cell culture plates were coated using the COAT-1 coating solution. iPSCs were dissociated into single cells, resuspended in warm DE diff Day 0 medium and seeded at a density of 3.0–4.0 × 10^4^ cells/cm^2^ (in 0.2 ml medium/cm^2^). On day 1, 2, 3, 4 and 6, medium changes were performed using warm DE diff medium designated for the specific day. On day 7, the DE cells were enzymatically dissociated and further differentiated using the Cellartis Hepatocyte Differentiation Kit. In brief, DE cells were seeded in Hepatocyte Thawing and Seeding Medium at an initial density of 1.2–1.3 × 10^5^ cells/cm^2^ in 24‐well plate with 1.0 ml of medium/well. The DE cells were differentiated in Hepatocyte Thawing and Seeding Medium for 2 days at 37 °C, before changing to Hepatocyte Progenitor Medium for another 5 days of differentiation to hepatoblasts. The cells were then further differentiated in Hepatocyte Maturation Medium for 4 days and finally matured in Hepatocyte Maintenance Medium for up to 14 days. Medium was exchanged every 2–3 days following the manufacturer’s instructions. Hepatocyte differentiation conditions were kept identical for all the iPSC lines except for some lines (B1, L1 and W2) in which cell seeding density for DE induction needed minor adjustments.

### 4.3 Compound treatment

Cells differentiated on day 23 were treated for 24 h with 3.0 mM DGA or 10.0 μM UA. For each treatment condition, cells from two wells of a 24-well plate were pooled to generate one sample, and two replicate samples (n = 2) were generated from separate wells of the same differentiation plate. The concentrations for DGA and UA were selected based on the cytotoxicity results reported previously [5], which showed an approximately 10% average decrease in cell viability across the cell lines. For DGA, a stock solution of 80 mM was prepared fresh in maintenance medium immediately before exposure. The stock solution was further diluted to the final treatment concentration (3.0 mM) using maintenance medium.. For UA, a stock solution of 20 mM was prepared in DMSO and stored at −20 °C until use. Immediately before cell treatment, the stock solution was diluted 2000 × with maintenance medium to yield a treatment solution containing 10.0 μM UA and 0.05% (v/v) DMSO. Cells treated with maintenance medium alone were used as the negative control for both DGA and UA treatments. Previous *in vitro* validation studies have shown that DMSO concentrations of up to 0.5–1.0% result in minimal background variation in gene expression [18]. Given the low final concentration of DMSO [0.05% (v/v)] in the UA treatment solution, its contribution to the observed transcriptomic responses is expected to be limited.

### 4.4 Total RNA extraction and quality control

Total RNA samples were prepared from control and treatment cells and quality checked as described previously [19]. Briefly, cells were lysed in RLT buffer (Qiagen, Valencia, CA) supplemented with 1% (v/v) β-mercaptoethanol at the end of the exposure and the lysates were stored at −80 °C until RNA extraction. The lysates were thawed on ice and homogenized using QIAshredder (Qiagen). Total RNA was purified from the cell lysates using EZ1 Advanced XL automated RNA purification instrument (Qiagen) with the EZ1 RNA Cell Mini Kit (Qiagen), and instructions from the manufacturer were followed. An on-column DNase digestion step was included to remove contaminating genomic DNA. Total RNA concentration and purity (260/280) were subsequently measured using a NanoDrop 2000 UV–vis spectrophotometer (NanoDrop Products, Wilmington, DE). RNA quality was further checked using the Agilent 2100 Bioanalyzer (Agilent, Santa Clara, CA) with the RNA 6000 Nano Reagent Kit (Agilent) to obtain the RNA integrity number (RIN).

### 4.5 RNA sequencing and data analysis

Sequencing libraries were prepared using the Illumina Stranded mRNA Prep, Ligation kit (Illumina, San Diego, CA) following protocols provided by the manufacturer. Briefly, messenger RNAs (mRNAs) with polyA tails were purified from 100 ng of total RNA of each sample using oligo(dT) magnetic beads then fragmented and denatured. First strand complementary DNA (cDNA) was synthesized by reverse transcription of the hexamer-primed RNA fragments. Then a second strand cDNA was synthesized to replace the RNA template and generate blunt-ended, double-stranded cDNA fragments. In place of deoxythymidine triphosphate (dTTP), deoxyuridine triphosphate (dUTP) was incorporated to quench the second strand during amplification and achieve strand specificity. An adenine (A) nucleotide was added to the 3ʹ ends of the blunt fragments to prevent them from ligating to each other during adapter ligation. A corresponding thymine (T) nucleotide on the 3ʹ end of the adapter provides a complementary overhang for ligating the adapter to the fragment. Pre-index anchors were ligated to the ends of the double-stranded cDNA fragments to prepare them for dual indexing, and a subsequent PCR amplification step added the index adapter sequences. The adapter-ligated fragments were purified using magnetic beads. In the next step, a 13-cycle PCR was run to selectively amplify the anchor-ligated DNA fragments and add indexes and primer sequences for cluster generation. The resulting product is a dual-indexed library, i.e., DNA fragments with adapters at each end. Finally, the dual-indexed libraries were purified using magnetic beads.

The libraries were analyzed using the Agilent 2100 Bioanalyzer and DNA 1000 Kit for size distribution and further quantified by Qubit Flex Fluorometer using the Qubit dsDNA BR Assay Kit (Thermo Fisher Scientific). The library of each sample was diluted to 2 nM in resuspension buffer (RSB) and 10 µl of the diluted libraries from all the samples were pooled. The pooled library is further diluted to 750 pM in RSB with Tween and 20 µl was loaded onto a thawed P3 200-cycle cartridge. Next, paired-end RNA-seq cluster generation and sequencing by synthesis was performed on an Illumina NextSeq 2000 instrument, following protocols provided by the manufacturer. Depths of an average ∼17 million paired 75 bp reads were generated for each sample. Concatenated base call files (.cbcl) generated from the sequencing run were converted to a FASTQ file per sample by the DRAGEN BCL Convert v3.8.4 pipeline. The .cbcl files were further analyzed by the DRAGEN RNA pipeline to generate transcript quantification results at the gene level (.quant.genes.sf). The human reference genome hg38-alt-aware was used for the alignment.

Gene-level counts obtained for each sample were subsequently normalized by DESeq2 median-of-ratios method in Partek Flow (Partek; Chesterfield, MO). Subsequent unsupervised data exploration, including principal component analysis (PCA), were also carried out in Partek Flow (Partek). Genes with a maximum normalized count ≤10 across all samples were excluded from analysis.

## Limitations

The dataset includes only three ethnic groups, with three cell lines in each group, which may limit its ability to capture the variability of the broader human population. Only two replicate samples from the same differentiation experiment were included in each treatment group, which limits the statistical power of the dataset for drawing robust conclusions regarding variability across ethnicities and treatment conditions. In addition, only cell lines from male donors were used; therefore, sex-related differences were not assessed. Differences in the ages of the donors (0–27 years) from whom the cell lines were derived may have contributed additional variability to the dataset. Furthermore, the nonuniform hepatocyte differentiation efficiency among the nine iPSC lines observed in the previous study [5] may have introduced bias into the dataset. Finally, a matched DMSO vehicle control was not included for the UA treatment; therefore, potential effects of the low concentration of DMSO [0.05% (v/v)] on gene expression cannot be completely excluded.

## Ethics Statement

The authors have read and follow the ethical requirements for publication in *Data in Brief*. The current work does not involve human subjects, animal experiments, or any data collected from social media platforms.

## Credit Author Statement

**Xiugong Gao:** Conceptualization, Methodology, Investigation, Formal analysis, Visualization, Supervision, Project administration, Writing – original draft, Writing – review & editing. **Miranda R. Yourick:** Investigation, Formal analysis, Visualization. **Rong Li:** Methodology, Investigation, Formal analysis, Writing – review & editing. **Kayla Campasino:** Investigation, Formal analysis, Writing – review & editing. **Robert L. Sprando:** Conceptualization, Resources, Funding acquisition. **Jeffrey J. Yourick:** Conceptualization, Resources, Funding acquisition.

## Data Availability

GEOTranscriptomic comparison of hepatocytes derived from a panel of human induced pluripotent stem cells with different ethnicities (Original data).
